# A New Threat to Conifer Cones: *Cydia kamijoi* (Oku, 1968) (Lepidoptera, Tortricidae), a New Record for China, Based on Morphological and DNA Barcoding Analyses

**DOI:** 10.3390/insects16050485

**Published:** 2025-05-02

**Authors:** Niya Jia, Fang Niu, Xiaomei Wang, Defu Chi, Jia Yu

**Affiliations:** The Key Laboratory of Sustainable Forest Ecosystem Management-Ministry of Education, College of Forestry, Northeast Forestry University, Harbin 150040, China; jianiya9937@126.com (N.J.); 15944205798@163.com (F.N.); xiaomeiwang930425@outlook.com (X.W.)

**Keywords:** *Cydia kamijoi*, DNA barcode, COI, new record, species identification

## Abstract

*Cydia kamijoi* (Oku, 1968) (Lepidoptera, Tortricidae) is a pest of conifer cones. It was first found in Hokkaido, Japan and was considered to be an endemic species of Hokkaido, which was rarely reported. Here, we report *C. kamijoi* in China for the first time, whose larvae feed on *Pinus koraiensis* pine cones. Descriptions of the larval and adult morphology of C. kamijoi, along with the COI DNA barcoding data available and the phylogenetic analysis are provided for this species for the first time. The emergence of *C. kamijoi* has severely threatened the health of *P. koraiensis* cones. This work may have important implications for the pest control of *P. koraiensis* cones in Northeast China.

## Simple Summary

The genus *Cydia* (Lepidoptera: Tortricidae), a taxon of significant entomological interest, encompasses approximately 255 species with a cosmopolitan distribution. Within the geographic confines of China, 15 species within this genus have been documented, to date. Through comprehensive morphological analysis, the present study formally documents *Cydia kamijoi* (Oku, 1968) as a newly recorded species within the Chinese fauna, thereby expanding the known diversity of the genus in this region.

## 1. Introduction

The genus *Cydia* was erected by Hübner ([1825]: 375) and contains approximately 255 currently described species that are distributed worldwide [1], and the type species is *Phalaena pomonella* (Linnaeus 1758: 538). The systematic classification and nomenclature of the genus *Cydia* have a complex history [2,3,4]. *Cydia* is the currently accepted generic name for pomonella and congeneric species [5,6,7,8,9,10].

To date, there are 15 species of *Cydia* distributed in China, including *C. coniferana* (Saxesen), *C. curvivalva* Liu & Yan, *C. dalbergiacola* Liu, *C. glandicolana* (Danilevsky), *C. grunertiana* (Ratzeburg), *C. illutana* (Herrich-Schäffer), *C. kurokoi* (Amsel), *C. maackiana* (Danilevsky), *C. nigricana* (Fabricius), *C. pactolana* (Zeller), *C. pomonella* (Linnaeus), *C. splendana* (Hübner), *C. strobilella* (Linnaeus), *C. trasias* (Meyrick), and *C. zebeana* (Ratzeburg), among which nine species can be found in Heilongjiang province [11,12]. This genus contains many pest species, and important pests of fruit, nuts, cones, and pods in the world [13]. *Cydia* species have been a focal point of research concerning potential issues with coniferous tree pests [14,15,16,17].

In addition to traditional morphological methods, molecular taxonomy has been proposed as a method for accurate species identification [18,19]. DNA barcoding is a species identification method that uses a standardized segment of the mitochondrial cytochrome c oxidase I gene (mtDNA COI) [20,21]. With the progressive development of molecular methods, currently the database of the National Center for Biotechnology Information (NCBI) records more than 800 mtDNA COI sequences of *Cydia*. Hu et al. [22] confirmed through phylogenetic reconstruction that the genus *Cydia* in a broad sense in the traditional classification is actually a polyphyletic complex, whose members include Holarctic species of *Cydia*, Afrotropical species of *Fulcrifera* (although the latter includes several Palaearctic species not included in our analysis), and *Leguminivora* (from Asia, Australia, and Africa), forming a non-monophyletic cluster. Within the genus *Cydia*, there is a significant pattern of ecological differentiation: large monophyletic clades with Pinaceae as hosts were resolved in phylogeny, such as *C. piperana*, *C. toreuta*, *C. ingens*, *C. pactolana,* and *C. coniferana*, which not only have highly conserved mitochondrial COI sequence characteristics, but also show convergent evolution at the morphological level—these species all show a dark forewing ground color with a pair of nearly parallel raised metallic bands [23].

In this paper, we report a new record of *Cydia* from China. *C. kamijoi* appeared in northeast China for the first time and then became one of main pests of *Pinus koraiensis* cones. We redescribe the adult and larval morphological characteristics of *C. kamijoi* and provide the DNA barcoding information and phylogenetic placements for the first time. Implications of this new threat to the forestry economy are discussed.

## 2. Materials and Methods

### 2.1. Collecting Information

The specimens were collected in Linkou (45.5649° N, 130.1963° E), Heilongjiang province, China between 20 July 2021 and 25 August 2021. Damaged cones were collected from 50-year-old pine trees (*Pinus koraiensis*). Those damaged cones were transported to the laboratory and the insects found on them were reared at room temperature conditions until adults emerged, which were captured and pinned. Parts of the cones were dissected to collect larvae, and the larvae were placed in 95% ethanol at −20 °C and identified using morphology and DNA barcodes. Voucher specimens were preserved at Entomological Specimen Room of Northeast Forestry University (NEFU) in Harbin, China, and are available on request. The relevant experiments and analyses were conducted at NEFU.

### 2.2. Morphological Study

The abdomens of adults were boiled in 10% NaOH solution, cleaned, and the genitalia were dissected in distilled water. Photographs were taken using a Canon A620 camera (Canon, Tokyo, Japan) mounted on the stereomicroscope. All photographs and images were processed with Adobe Photoshop CS6 v. 13.0.

### 2.3. DNA Extraction, Amplification, and Sequencing

Three larvae and six adults (two males and four females) were used for molecular analysis using the standard mtCOI DNA barcodes. The total DNA was extracted from each adult hind leg and whole larvae by using the TIANamp Micro DNA Kit (TIANGEN, Beijing, China) according to the manufacturer’s specifications. The mitochondrial COI gene was amplified by PCR using the following primer pair designed for Lepidoptera: LEP-F1, 5′-ATTCAACCAATCATAAAGATAT-3′; and LEP-R1, 5′-TAAACTTCTGGATGTCCAAAAA-3′ [24]. The PCR reaction was performed in a total 25 µL volume: 0.5 µL DNA, 1 µL of each primer (100 ng/µL), 12.5 µL of Takara Ex Taq DNA polymerase (Takara Biomedical Technology, Beijing, China) and 10 µL nuclease-free water. PCR conditions were as follows: initial denaturation at 95 °C for 2 min, 35 cycles of denaturation at 95 °C for 30 s, annealing at 49 °C for 30 s, extension at 72 °C for 30 s, with a final extension at 72 °C for 5 min. All PCR products were sequenced by Sangon Biotech Company (Shanghai, China).

### 2.4. Sequence Processing and Phylogenetic Analyses

The nine resulting sequences were evaluated using the Chromas v2.6.5 and compared against homologous sequences deposited in the NCBI (www.ncbi.nlm.nih.gov/Genbank, URL (accessed on 23 June 2022)) and BOLD (www.barcodinglife.org, URL (accessed on 23 June 2022)) databases; all sequences were submitted to GenBank and accession numbers were obtained. In addition, five *Cydia* and eight non-*Cydia* species of Olethreutinae sequences were downloaded from GenBank, and *Pandemis heparana*, *Aethes cnicana* and *Gynnidomorpha alismana* were used as the outgroup. The GenBank accession numbers of all sequences analyzed in this study are listed in Table 1. The sequences were aligned using Clustal W and sequence genetic distances were estimated by the Kimura 2 parameter model (K2P) implemented on MEGA v7.0 [25], and the final aligned dataset included 30 COI sequences with 658 bp. The phylogenetic analysis of the concatenated-sequence matrix was conducted using Maximum likelihood (ML) and Bayesian inference (BI) methods. The ML analysis was constructed by GTR+I+G nucleotide model with 1000 bootstrap replicates using MEGA v7.0. The BI analysis was constructed by MrBayes v3.2.6 which uses Markov chain Monte Carlo (MCMC) sampling algorithms. The nucleotide substitution model used was GTR+I+G [26], run for 1,000,000 generations and sampled every 1000 generations, using four chains. The first 25% trees were discarded as burn-in, and the remaining trees were used to calculate posterior probabilities (pp). The phylogenetic trees were edited in FigTree v1.4.2.

## 3. Results

### 3.1. Species Descriptions

Family Tortricidae Latrielle, 1803

Subfamily Olethreutinae Walsingham, 1895

Tribe Grapholitini Guenée, 1845

Genus *Cydia* Hübner, [1825]

*Cydia kamijoi* (Oku, 1968)

*Cydia kamijoi* Oku, 1968 (Laspeyresia), Konty 36: 235. Type locality: Japan, “Hokkaido, Kitami”. Holotype: EIHU. female.

Material examined. Two males, four females, three larvae of unknown gender, Linkou County, Heilongjiang Province, China, 45.5649° N; 130.1963° E, 20.VII-25VIII.2021, leg. Niya Jia, laboratory, in *Pinus koraiensis* cones.

**Figure 1:**
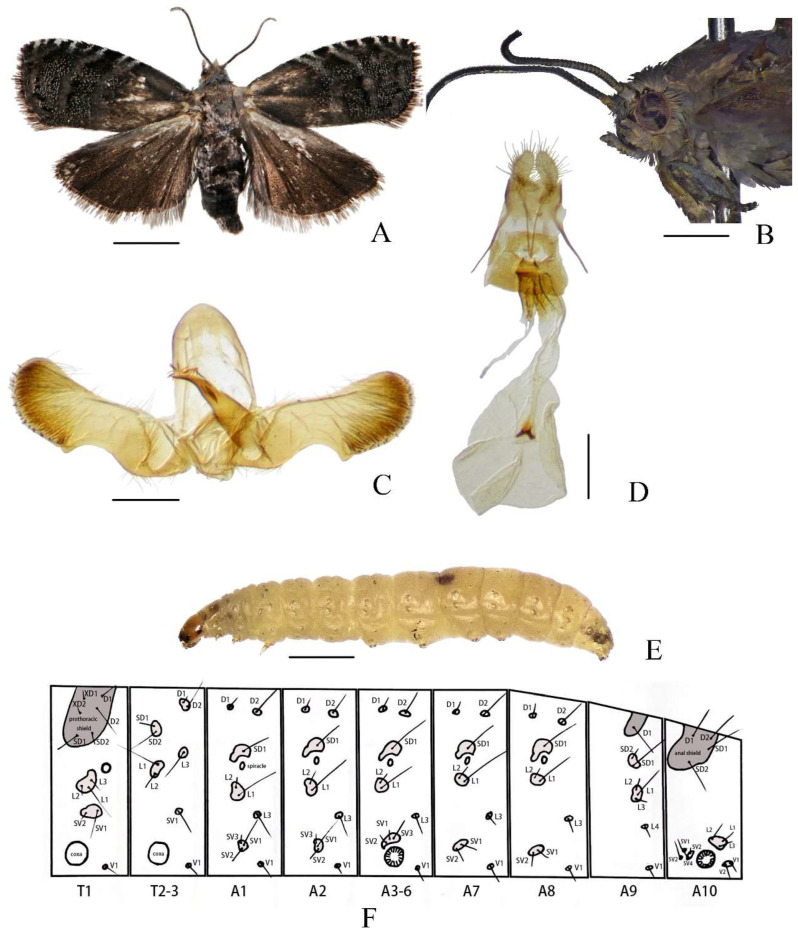
*Cydia kamijoi* Oku, 1968. (**A**) adult; (**B**) adult head; (**C**) male genitalia; (**D**) female genitalia; (**E**) larva; (**F**) chaetotaxy. Scale bars: 6 mm (**B**); 2 mm (**A**); 1 mm (**E**); 0.1 mm (**C**,**D**).

Diagnosis. In adults (Figure 1A), *Cydia kamijoi* is completely black without clear patterns or spots; several other black species have white spots or patterns. In male genitalia (Figure 1C), tegumen elliptical, relatively narrow on top; sacculus angle blunt; cucullus nearly rectangular. In female genitalia (Figure 1D), lamella, postvaginalis somewhat chitinized.

Description. Head (Figure 1A,B): Head brown covered with grey scales. Antenna grayish black, filiform, with dense, short, dark brownish grey cilia. Ocellus glossy yellow. Labial palpus greyish mixed with light brown.

Thorax (Figure 1A,B): Thorax brown covered with grey scales. Wingspan 12–13 mm. Forewing apex obtusely rounded, ground color dark brownish grey with sparse white scales, brown at base costa with eight pairs of silver-whitish strigulae from costal 1/3 to apex three blue-whitish metallic striae arranged between basal 1/3 of forewing and outer margin terminal line distinct narrowly black cilia blackish brown. Hindwing dark blackish brown, apex distinctly darker than base cilia dark grey. Legs bronzy grey.

Abdomen (Figure 1A,C,D): Abdomen dark brown covered with greyish white scales (Figure 1A). Male genitalia (Figure 1C), tegumen elliptical, relatively narrow on top. Uncus and socii degenerate. Valva elongate, neck distinct smooth, rounded apically; basal opening broad and subrectangular, concave; sacculus angle blunt; cucullus nearly rectangular, with numerous long hairs inwardly and short spines along the ventral and terminal margins, slightly protuberant at ventral corner. Aedeagus stout, short, point-ed apically; vesica with 18–20 cornuti. Female genitalia (Figure 1D), papillae anales elliptical, elongated, hairy. Apophysis posterioris longer than apophysis anterioris. Lamella postvaginalis somewhat chitinized. Ostium bursae circular, narrowed anteriorly. Ductus bursae short, sclerotized. Tubular part of bursae nearly transparent. Corpus bursae circular, with two small coniform signa, united with each other at base.

Larva. Late instar larvae approximately 8–11 mm long, abdomen cream-colored, translucent. Head and pronotum brown. Legs white, with uniordinal crochets (Figure 1E). Pinaculum small, slightly darker than body. Chaetotaxy as shown in Figure 1F.

Prothoracic Segment (T1): The prothoracic segment (T1) bears the following setae: one pair of prothoracic setae (XD1 and XD2); one pair of dorsal setae (D1 and D2); one pair of subdorsal setae (SD1 and SD2), six setae (XD1, XD2, D1, D2, SD1, and SD2) are situated on a large pinacula, with XD2 and D2 positioned ventrad to XD1 and D1, respectively; three lateral setae (L1, L2, and L3) are clustered on a single pinacula; two subventral setae (SV1 and SV2) share a common pinacula.

Mesothoracic (T2) and Metathoracic (T3) Segments: The mesothoracic (T2) and metathoracic (T3) segments exhibit nearly identical chaetotaxy: the dorsal pinacula bears one pair of dorsal setae (D1 and D2); one pair of subdorsal setae (SD1 and SD2); three lateral setae (L1, L2, and L3), L1 and L2 are clustered on a single pinacula, while L3 is positioned separately.

Abdominal Segments (A1–A10):

A1 and A2: Abdominal segments I (A1) and II (A2) exhibit nearly identical chaetotaxy. Each segment bears one pair of dorsal setae (D1 and D2), a single subdorsal seta (SD1), and three lateral setae (L1, L2, and L3). Among the lateral setae, L1 and L2 are clustered on a shared pinacula, while L3 is positioned separately. Three subventral setae (SV1, SV2, and SV3) coalesce on a single pinacula.

A3–A6: Segments III–VI (A3–A6) share identical setal arrangements: one pair of dorsal setae (D1 and D2), a single subdorsal seta (SD1), three lateral setae (L1–L3), and three subventral setae (SV1–SV3).

A7 and A8: Segments VII (A7) and VIII (A8) display similar chaetotaxy, each bearing one pair of dorsal setae (D1 and D2), a single subdorsal seta (SD1), three lateral setae (L1–L3) with L1 and L2 clustered on a pinacula and L3 positioned separately, and two subventral setae (SV1 and SV2).

A9: Segment IX (A9) is characterized by a single dorsal seta (D1), one pair of subdorsal setae (SD1 and SD2), four lateral setae (L1–L4) with L1–L3 clustered on a pinacula, and L4 positioned separately.

A10: Segment X (A10) bears one pair of dorsal setae (D1 and D2) and one pair of subdorsal setae (SD1 and SD2), all coalesced on a shared pinacula. Three lateral setae (L1–L3) are clustered on a single pinacula, while four subventral setae (SV1–SV4) are present, with SV3 and SV4 sharing a pinacula. Two ventral setae (V1 and V2) are positioned on a common pinacula. The larval posterior end lacks an anal fork.

Distribution. China (Heilongjiang, new record), Korea (Jeju) and Japan (Hokkaido).

Ecology. The following life history information is summarized from our data. *C. kamijoi* has one generation per year in Northeast China. Adults emerge from the end of July to the end of August. Observed indoors and outdoors, the mature larvae leave the cones between September and October and over winter in the soil.

Host. Cones of *Pinus koraiensis* (New to China), *Abies koreana* (Korea) and *Abies sachalinensis* (Japan) [27,28].

Remark. In Oku’s [27] report, there was a lack of morphological description of the male genitalia of *C. kamijoi*. Some morphological characters of Korean species illustrated by Shin et al. [28] are different from those of the Chinese and Japanese specimens, e.g., valva is slender and ventral edge of cucullus is sharp, and two signa not united at base. There is a possibility that the Korean species are not *C. kamijoi*.

### 3.2. Molecular Study

**Figure 2:**
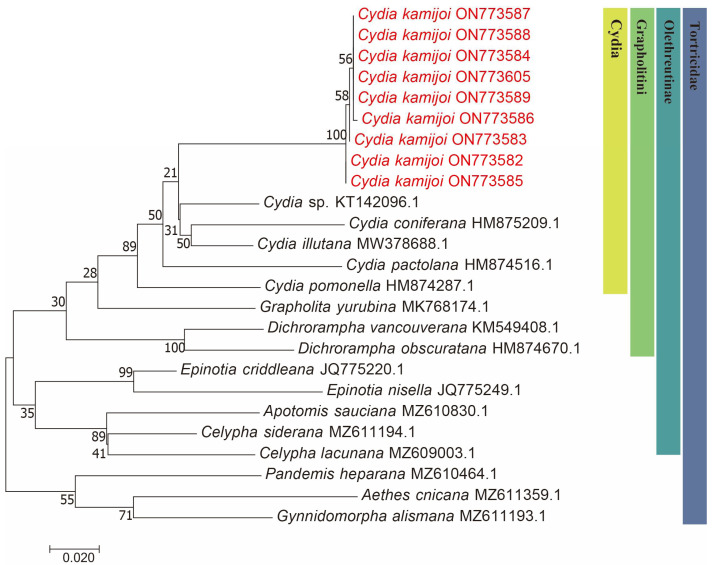
Maximum likelihood (ML) phylogenetic trees constructed based on mtDNA COI. The number at each branch indicates the percentage supported by bootstrap.

**Figure 3:**
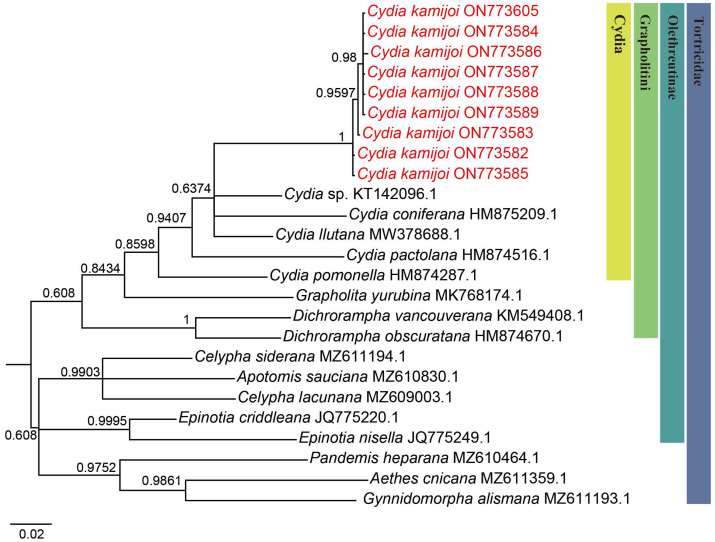
Bayesian inference (BI) phylogenetic trees constructed based on mtDNA COI. The number at each branch indicates posterior probability.

We successfully obtained 658 bp fragments of COI for nine *Cydia* species (three larvae, two adult males and four adult females) and sequenced them (the GenBank accession numbers are provided in Table 1). The final dataset included 25 COI sequences representing 17 species of 9 genera in Tortricidae (Table 1). Both maximum likelihood (ML) (Figure 2) and Bayesian inference (BI) (Figure 3) analyses of the COI dataset showed similar results. The monophyly of the *C. kamijoi* is supported by bootstrap values (100% in both ML and BI). The intraspecific divergences within nine *C. kamijoi* specimens were detected as extremely low (0–0.5%). Four haplotypes, the base composition of sequences, was highly biased toward TA (67.6%), as usually observed in insect mitochondrial genomes. The smallest interspecific divergence (mean = 7.4%) was observed between *C. kamijoi* and *Cydia* sp. (KT142096.1). Interspecific genetic distances between *C. kamijoi* and other species of *Cydia* ranged from 7.2% to 10.9%, and the genetic distance between *C. kamijoi* and Tortricidae family is 11.5%~15.3%. The results are in accordance with the patterns described for other Lepidoptera families: Hebert [21] proposed that the accepted threshold to delimit species according to COI barcode sequences is 3%. The intraspecific genetic divergences are, in general, lower than 2% in the Tortricidae [29,30,31]; DNA variations observed within *C. kamijoi* specimens in this study were lower than 1%. Overall, the molecular analysis supported the morphological identification results.

## 4. Discussion

This is the first record of *C. kamijoi* in China. We found the larvae in damaged cones, which were allowed to mature into adults. Both adults and larvae specimens were identified to species level by morphological and molecular methods. The morphological analysis confirmed that the adult specimens are *C. kamijoi* and larvae that belonged to the family Olethreutinae. The BLASTn results obtained from the NCBI database showed that the COI sequences of our specimens are *Cydia*. In this study, morphological characteristics of larvae and molecular information of species identification have provided, for the first time, information on the distribution and host species of *C. kamijoi*. In Oku’s [27] report, there was a lack of morphological description of the male genitalia of *C. kamijoi*. Female genitalia morphological characters of Korean species illustrated by Shin et al. [28] are different from those of the Chinese and Japanese specimens. There are disputes in the morphological characteristics of *C. kamijoi* in China, Korea, and Japan. We have provided the molecular identification information of *C. kamijoi* for the first time, providing important evidence for the identification of *C. kamijoi*. When morphological characteristics are not obvious or difficult to distinguish, by combining morphological and molecular biology methods, we can resolve controversial issues in insect identification.

When *C. kamijoi* was first found in Hokkaido, Japan [27] and was originally thought to be endemic to Japan. In recent years, it was found to occur in Korea [28] and China (this work). It is interesting that the distribution of *Cydia* centers in three countries around the sea of Japan, ranging between 33° E to 45° E and 126° N to 143° N, but the three sites are separated by ocean; it is seemingly a strange and isolated distribution of *C. kamijoi*. Hence, it cannot be judged whether it has been there for a considerable period or is a recent introduction. The possibility of transoceanic dispersal of insects has been discussed by various authors [32,33,34,35]. It is not possible to judge whether *C. kamijoi* is an invasive species based on the information currently available. In our study, the COI sequences of *C. kamijoi* were submitted to the NCBI Gene Bank; we still need more material and molecular information of *C. kamijoi* from different regions for further studies and to obtain a reasonable explanation for the dispersal history. The quantity of *C. kamijoi* has greatly increased over recent years in Northeast China and now forms a potential threat to cone production of an economically important pine tree. We need to continue to investigate about its hosts and harm in China as well as remain vigilant.

## Figures and Tables

**Table 1:** Samples of COI genes used in this study.

Subfamily	Tribe	Species	GenBank NO.
Olethreutinae	Grapholitini	*Cydia kamijoi* c1	ON773605
		*Cydia kamijoi* c2	ON773582
		*Cydia kamijoi* c3	ON773583
		*Cydia kamijoi* c4	ON773584
		*Cydia kamijoi* c5	ON773585
		*Cydia kamijoi* c6	ON773586
		*Cydia kamijoi* y1	ON773587
		*Cydia kamijoi* y2	ON773588
		*Cydia kamijoi* y3	ON773589
		*Cydia* sp.	KT142096.1
		*Cydia coniferana*	HM875209.1
		*Cydia illutana*	MW378688.1
		*Cydia pactolana*	HM874516.1
		*Cydia pomonella*	HM874287.1
		*Grapholita yurubina*	MK768174.1
		*Dichrorampha vancouverana*	KM549408.1
		*Dichrorampha obscuratana*	HM874670.1
	Olethreutini	*Apotomis sauciana*	MZ610830.1
		*Celypha lacunana*	MZ609003.1
		*Celypha siderana*	MZ611194.1
	Eucosmini	*Epinotia criddleana*	JQ775220.1
		*Epinotia nisella*	JQ775249.1
Tortricinae	Archipini	*Pandemis heparana*	MZ610464.1
	Cochylini	*Aethes cnicana*	MZ611359.1
		*Gynnidomorpha alismana*	MZ611193.1

## Data Availability

All data are available in the manuscript.

## Abbreviations

EIHU	Entomological Institute, Hokkaido University, Sapporo, Japan
NEFU	Northeast Forestry University, Harbin, China
